# Benefits and costs of home-based pulmonary rehabilitation in chronic obstructive pulmonary disease - a multi-centre randomised controlled equivalence trial

**DOI:** 10.1186/1471-2466-13-57

**Published:** 2013-09-08

**Authors:** Anne E Holland, Ajay Mahal, Catherine J Hill, Annemarie L Lee, Angela T Burge, Rosemary Moore, Caroline Nicolson, Paul O’Halloran, Narelle S Cox, Aroub Lahham, Rebecca Ndongo, Emily Bell, Christine F McDonald

**Affiliations:** 1 Alfred Health, 99 Commercial Road, Melbourne 3004, Australia; 2 Institute for Breathing and Sleep, Melbourne, Australia; 3 La Trobe University, 99 Commercial Road, Melbourne, Australia; 4 Department of Epidemiology and Preventative Medicine, Monash University, Melbourne, Australia; 5 Austin Health, Melbourne, Australia; 6 The University of Melbourne, Melbourne, Australia

## Abstract

**Background:**

Pulmonary rehabilitation is widely advocated for people with chronic obstructive pulmonary disease (COPD) to improve exercise capacity, symptoms and quality of life, however only a minority of individuals with COPD are able to participate. Travel and transport are frequently cited as barriers to uptake of centre-based programs. Other models of pulmonary rehabilitation, including home-based programs, have been proposed in order to improve access to this important treatment. Previous studies of home-based pulmonary rehabilitation in COPD have demonstrated improvement in exercise capacity and quality of life, but not all elements of the program were conducted in the home environment. It is uncertain whether a pulmonary rehabilitation program delivered in its entirety at home is cost effective and equally capable of producing benefits in exercise capacity, symptoms and quality of life as a hospital-based program. The aim of this study is to compare the costs and benefits of home-based and hospital-based pulmonary rehabilitation for people with COPD.

**Methods/Design:**

This randomised, controlled, equivalence trial conducted at two centres will recruit 166 individuals with spirometrically confirmed COPD. Participants will be randomly allocated to hospital-based or home-based pulmonary rehabilitation. Hospital programs will follow the traditional outpatient model consisting of twice weekly supervised exercise training and education for eight weeks. Home-based programs will involve one home visit followed by seven weekly telephone calls, using a motivational interviewing approach to enhance exercise participation and facilitate self management. The primary outcome is change in 6-minute walk distance immediately following intervention. Measurements of exercise capacity, physical activity, symptoms and quality of life will be taken at baseline, immediately following the intervention and at 12 months, by a blinded assessor. Completion rates will be compared between programs. Direct healthcare costs and indirect (patient-related) costs will be measured to compare the cost-effectiveness of each program.

**Discussion:**

This trial will identify whether home-based pulmonary rehabilitation can deliver equivalent benefits to centre-based pulmonary rehabilitation in a cost effective manner. The results of this study will contribute new knowledge regarding alternative models of pulmonary rehabilitation and will inform pulmonary rehabilitation guidelines for COPD.

**Trial registration:**

ClinicalTrials.gov: NCT01423227.

## Background

Chronic obstructive pulmonary disease (COPD) is characterised by exertional dyspnoea, reduced exercise tolerance, marked disability and frequent hospitalisation. The current economic burden of COPD to the health system is considerable, with the majority of costs attributable to hospital use [1,2]. International guidelines for managing COPD include referral to pulmonary rehabilitation [3], supported by compelling evidence that such programs deliver improvement in exercise capacity, reduction in breathlessness and improvement in health-related quality of life (HRQOL), irrespective of disease severity [4]. Pulmonary rehabilitation is also associated with a reduced frequency of acute exacerbations and hospital admissions [5]. Despite the strong evidence of its benefits, the proportion of people with COPD who receive pulmonary rehabilitation is very small, with estimates from developed countries suggesting that it is delivered to less than 5% of those who would benefit each year [6,7]. Of those people with COPD who are referred to pulmonary rehabilitation, 8% to 50% will never attend [8,9], while non-completion rates range from 10% to 32% [10,11]. The practical reasons for this lack of access include a shortage of programs, particularly in rural and regional centres, and an insufficient number of qualified health professionals. Patient-related barriers to attendance have also been identified, with travel and transport to centre-based programs being the most common obstacles to attendance in this disabled group [8,10-16].

In view of these barriers, the current model of a centre-based pulmonary rehabilitation program is not addressing the needs of many people with COPD who would benefit from this intervention. If new models are not found, uptake of these programs by patients with COPD will continue to be limited. Home-based, unsupervised pulmonary rehabilitation has been proposed as an alternative model which may enhance access and uptake while containing the rising costs of healthcare associated with COPD [17-20]. Direct comparison of home and centre-based pulmonary rehabilitation has been explored in four studies [17-20]. Home-based programs were found to be safe, with improvement in exercise tolerance and HRQOL and reduced symptoms demonstrated in both the home and hospital-based groups. However, only one study was a non-inferiority trial [18] and important differences between supervised and unsupervised groups may not have been detected in other studies. Of greater importance is that no studies of self-monitored pulmonary rehabilitation have tested an entirely home-based intervention. In previous studies participants in the home training groups were required to attend the hospital for self-management education [17,18] or weekly monitoring [19]. These approaches fail to overcome the transport and mobility-related barriers to attendance.

Population ageing and the increasing health care burden associated with chronic disease provide an imperative to allocate our limited resources effectively [21]. The variety of program models and staffing used for home-based rehabilitation in COPD suggests that costs could vary considerably. One home-based program incorporated frequent home visits from clinicians to supervise exercise [20], a model which has proved to be more costly than centre-based rehabilitation in patients with cardiac disease [22]. Self-monitored exercise programs utilising telephone follow up have demonstrated clinical effectiveness [18], but not all components of pulmonary rehabilitation were delivered at home and no cost comparison to centre-based programs was undertaken. Analysis of the costs of delivery is a critical determinant of whether such a model of care is suitable for implementation in clinical practice.

Comprehensive economic evaluation includes both costs to the healthcare service and the patient [23]. In addition, cost effectiveness analysis incorporates the evaluation of the impact of an intervention on the patient, to determine if benefits are accrued beyond standard healthcare gains. Such gains may be accessed through measurement of quality-adjusted life years (QALYs) which quantify the benefit gained due to an intervention by measuring the change in quality of life over time. In COPD, centre-based pulmonary rehabilitation (exercise training and education) has been demonstrated to be cost-effective with improvement in QALYs [24,25]. However, no previous studies have assessed such outcomes for home-based pulmonary rehabilitation.

There is a need for new models of pulmonary rehabilitation which allow all program components to be delivered at home, with proven clinical outcomes and low costs. The aims of this study are to 1) compare completion rates of a home-based self-monitored pulmonary rehabilitation program with a hospital-based, outpatient program in people with COPD; 2) compare the clinical benefits of home-based pulmonary rehabilitation and hospital-based pulmonary rehabilitation and 3) compare the costs of home-based and hospital-based pulmonary rehabilitation. We hypothesise that the proportion of patients who complete pulmonary rehabilitation will be greater in the home-based intervention; the clinical effects on exercise capacity, symptoms and quality of life will be equivalent in both models; and the home-based program will be more cost-effective.

## Methods

### Design

A randomised, controlled, equivalence trial at two tertiary hospitals (Austin Health and Alfred Health) in Melbourne, Australia.

### Participants

To be eligible for enrolment, participants must be greater than 40 years of age, with a diagnosis of COPD based on an FEV_1_/FVC ratio of < 70% [3] and a smoking history (current or former) of a minimum of 10 packet years. Participants will be excluded if they have 1) diagnosis of asthma, 2) attended a pulmonary rehabilitation program within the last 2 years, 3) experienced an exacerbation of COPD within the last four weeks or 4) have other comorbidities which will prevent participation in an exercise training program.

### Recruitment and randomisation

Potential participants will be identified via attendance at respiratory medicine clinics at the Austin or Alfred Hospitals, upon referral to pulmonary rehabilitation at these sites, or screened from the existing database of the pulmonary rehabilitation programs at each site. Eligible participants will be approached by the researchers who will explain the study and provide participants with written and verbal information. The Human Research Ethics Committees of Alfred Health, Austin Health, La Trobe University and Monash University have approved this study, and written, informed consent will be obtained from all participants. Participants will be randomised to the hospital or the home based group using stratified block randomisation, with stratification for site (Austin or Alfred) and disease severity (FEV_1_ < 50% predicted versus FEV_1_ > 50% predicted) [3]. Randomisation will be undertaken using a computer generated sequence and allocation will be concealed using sealed, opaque envelopes. Participants will be allocated to groups after completion of baseline assessment. The flow of participants through the study will reflect the recommendations of the Consolidated Standard of Reporting Trials (CONSORT) [26].

### Interventions

#### Hospital-based pulmonary rehabilitation program

Participants will undertake a twice weekly, 8-week outpatient group-based supervised program, with individually prescribed exercise training and self-management education, to be undertaken at their site of recruitment. The program will follow the guidelines for exercise prescription in COPD [27]. This will include 30 minutes of aerobic exercise at each session, as well as upper and lower limb strength training, which will be comprised of both functional tasks and free weight training. The initial aerobic exercise prescription for walking will be set at 80% of the speed walked during a 6-minute walk test (6MWT) and at 60% of the maximal work-rate for cycling [28] and progressed each week according to patient symptoms. Resistance training will utilize functional activities such as stair training and sit to stand from a chair. Participants will be monitored with pulse oximetry during exercise training. Participants will also be encouraged to exercise at a moderate intensity at home an additional three times per week [27] and this activity will be recorded in a home diary which is reviewed weekly. Skill-orientated education and training sessions conducted by members of the multi-disciplinary team will be provided, with topics consistent with current recommendations [27,29]. A record of sessions attended by each participant will be maintained. The delivery of the pulmonary rehabilitation is standardised across the two sites following completion of previous collaborative pulmonary rehabilitation trials [30].

#### Home-based self-monitored pulmonary rehabilitation program

##### Exercise training

Participants will undertake an aerobic and strength training program of similar intensity and duration to the hospital program. The initial walking speed will be set at 80% of the speed walked during a 6-minute walk test (6MWT). A goal for walking distance will be set and distance will be recorded using a pedometer [31]. Participants will be encouraged to exercise for 30 minutes, five times per week and to record the completion of this activity in a home diary. Resistance training for the arms and legs will utilise daily activities and equipment that is readily available in the home environment (e.g. step-ups on an internal or external step, sit to stand from a standard height chair, water bottles for upper limb weights). To ensure safety and understanding of this exercise program, the initial exercise prescription will be established during a home visit by a physiotherapist. During this visit, elements of the self-management program that cannot be adequately discussed by subsequent telephone follow up will also be reviewed, including review of inhaler technique.

Participants will then be contacted by a physiotherapist each week by telephone for seven weeks to review the home diary, progress the exercise prescription and deliver self-management training. Disease specific self management training and exercise progression will be achieved using the principles of motivational interviewing [32,33]. The aims of this training are to advance disease knowledge, promote problem solving and facilitate health behaviour change [34]. Structured telephone modules will be used, with written prompts for the interviewer to explore and build motivation for change, then move towards commitment and action [32]. Exercise goals will be discussed and documented each week in the home diary. Participants will also be provided with a menu of topics covering other aspects of self management that are routinely discussed in a pulmonary rehabilitation program [29], including recognition and treatment of acute exacerbations; medication use; home exercise and physical activity; managing breathlessness; good nutrition; smoking cessation; and accessing community supports. Participants will be encouraged to choose one topic that is relevant to them for discussion and goal setting each week. Health goals will be documented in the standardized home diary. Training in self-management of acute exacerbations is considered a key element of pulmonary rehabilitation [29] and this topic will be discussed at least once during the program. Consistent with the principles of motivational interviewing, collaborative discussions will be directed toward building the perceived importance of behaviour change, increasing confidence, identifying motivations for change and setting specific goals though emphasis on participants producing their own arguments for change [32]. The physiotherapists undertaking the intervention will complete formal training in motivational interviewing and receive followup support from an experienced motivational interviewing practitioner.

At the completion of the 8-week intervention period, participants of both groups will be encouraged to continue with their home exercise program according to the previously outlined frequency and will be instructed to document their exercise participation in a monthly diary. Usual practice at both trial sites is to offer participants the opportunity to be involved in a community-based maintenance pulmonary rehabilitation program, in line with national standards [27]. Participants in both groups will be offered this option and the proportion of participants that accept and attend will be documented.

### Outcome measures

Baseline demographics of age, gender, body mass index and lung function (spirometry) will be collected. Spirometry will be repeated at 12 months to determine stability of lung function. Completion rates will be collated at the end of the intervention period and compared between the hospital and home-based groups, with an *a priori* definition of completion as undertaking a minimum of 70% of planned pulmonary rehabilitation sessions [35].

Clinical measures will be recorded at baseline, immediately following the intervention period (9 weeks) and at 12 months post completion of the intervention. An independent assessor, blinded to group allocation, will undertake the following measurements at each time point following the intervention period.

1) Functional exercise capacity will be measured using the 6-minute walk distance (6MWD), which is a valid measure of exercise capacity in COPD and is responsive to pulmonary rehabilitation [36]. Two tests will be conducted on each testing occasion using a standardised protocol [36], with the greatest distance recorded.

2) Dyspnoea will be measured using the Modified Medical Research Council Scale [37].

3) Disease-specific HRQOL will be measured using the self-reported Chronic Respiratory Disease Questionnaire (CRDQ), a valid and responsive tool in pulmonary rehabilitation [38]. It is comprised of 20 questions and assesses the domains of dyspnoea, fatigue, emotional function and mastery according to a 7-point Likert scale, with a higher score indicating better HRQOL.

4) A preference-based measure of HRQOL will be derived from the Short-form 36 (SF-36), a generic quality of life measure which is validated in COPD [39]. Responses to SF-36 will be used to classify health states using the SF-6D, for economic analyses [40].

5) Self efficacy will be measured using the Pulmonary Rehabilitation Adapted Index of Self-efficacy (PRAISE), a tool derived from the General Self-efficacy scale [41]. It is composed of 15 statements, each of which is scored from 1 to 4, with a higher score indicating greater levels of self efficacy. It is reproducible and sensitive to change following pulmonary rehabilitation in individuals with COPD [41].

6) Anxiety and depression will be measured with the Hospital Anxiety and Depression Scale (HADS). This questionnaire is designed to detect and measure the severity of anxiety and depression [42]. It consists of a series of 14 statements, with responses based on a 4-point Likert scale. The HADS is self-administered and has demonstrated responsiveness to pulmonary rehabilitation [43]. A higher score is indicative of greater anxiety or depression.

7) In a subgroup of participants, physical activity will be measured objectively using the SenseWear MF Armband (SWA, Bodymedia, Pittsburgh, USA) for a period of seven days. The SWA is a tri-axial accelerometer worn on the back of the left upper arm. The SWA produces estimates of energy expenditure and physical activity intensity using a proprietary algorithm (version 7.0), as well as measured outputs (step count). This monitor has been validated for assessment of daily activity [44] and used to monitor physical activity participation [45,46] in people with COPD. Variables to be reported are total wear time, number of steps per day, total energy expenditure per day, metabolic equivalents (METs) per day, awake sedentary time and time spent in moderate and vigorous activities.

### Economic evaluation

Economic evaluation will include direct (health system) and indirect (personal) health care costs related to the interventions and the 12 month follow up period. Direct costs will include staff time, consumables, communications, overheads and staff transportation. Information on the costs of the intervention will be based on detailed information on staff inputs by duration, type and resource use. Personal costs to individuals will include transportation, travel time, and impact of the intervention on the economic activities of other household members. Health system costs will include the costs of the intervention(s) and the costs associated with the use of health services, such as visits to the general practitioner, specialist visits, emergency department visits and hospitalization in the 12 month follow up period.

Participants will be contacted by telephone every month during the year-long followup period to document self-reported health care utilization and to encourage completion of the monthly diary. Out of pocket medical expenses will be recorded. Hospitalisation data and use of hospital services will be confirmed by medical record audit at the end of the trial. Medical and pharmaceutical resource use will be confirmed directly via Pharmaceutical Benefits Scheme (PBS) and Medicare Benefits Schedule (MBS) data. Medical services, government-subsidised pharmaceuticals and hospitalisation will be valued according to costs derived from the MBS, PBS and the Australian Refined Diagnosis Related Groups. Costs under the two interventions will be compared to assess the incremental impact of home-based rehabilitation for health system and personal costs. Sensitivity analyses will be carried out using different assumptions about the cost of travel and other personal healthcare costs in people with COPD.

To determine an individual’s self-valuation or utility of health state at a specific point in time, the SF-36 will be used to classify health states using the SF-6D [40]. This reflects six dimensions of health state including physical functioning, role limitations, social functioning, pain, mental health and vitality. Each attribute contains four to six levels that account for the 18,000 unique health states captured by the SF-6D. These indices are combined to provide a single preference utility index. Responses will be weighted by predetermined values established from the general population [47], with the highest score being 1.0 (best health state) and the lowest score being 0.0 (worst health state) [40] using the standard gamble utility measurement. This score will be converted to QALYs, which have the ability to simultaneously capture information about the gains achieved from reduced morbidity (quality gains) and reduced mortality (quantity gains) [23] and have the assumption that the duration of each status is exactly one half of the time between two measurement intervals. An incremental cost-effective ratio will compare the differences in costs with differences in gains in QALYs between the two interventions.

### Analysis

Continuous variables will be examined with repeated measures analysis of variance and linear mixed models, as appropriate. The proportion of participants who complete the program will be compared between groups using a chi-squared test and the relative risk of non-completion will be determined. All data will be analysed by intention to treat. A per-protocol analysis will also be conducted to reduce the risk of Type 1 error, as recommended in the CONSORT Extension for reporting of non-inferiority and equivalence trials [48]. Alpha will be set at 0.05.

Sample size calculations are based on the primary outcome of change in 6MWD. If there is truly no difference in the change in 6MWD between home-based and hospital based groups, then 144 participants are required to be 80% sure that the 95% confidence interval will exclude a difference in means of more than 25 metres. We have recently demonstrated that this is the minimal important difference for 6MWD in our population of patients with COPD [49]. This assumes a standard deviation of the change in 6MWD of 51 metres [50]. On the basis of our previous trials in pulmonary rehabilitation [49,50], we expect 15% attrition from the study; we will therefore randomize a total of 166 participants.

This sample size will also provide appropriate power for the secondary outcomes:

***Completion:*** Data from our centres indicate that only 66% people who are referred to pulmonary rehabilitation take up the referral and complete the centre-based program. Previous studies have documented very high completion rates for home-based programs of over 90% [18]. Using a more conservative estimate of 85% completion, 144 patients will be required to detect a difference in completion rates between hospital and home-based groups (72 in each group).

***Dyspnoea:*** If there is truly no difference in the change in the dyspnoea domain of the CRQ between home-based and hospital based groups, then 142 participants are required to be 80% sure that the 95% confidence interval will exclude a difference in means of more than 2.5 points. This is the minimal important difference for the dyspnoea domain of the CRQ [51] and assumes a standard deviation of the change in CRQ dyspnoea of 5.1 points [50].

## Discussion

This research will be the first study comparing both the clinical effects and cost effectiveness of delivering pulmonary rehabilitation in the home and hospital settings. With the substantial morbidity and mortality associated with COPD [2], and the growing proportion of people with COPD, there is an increasing need for cost effective treatment strategies. Pulmonary rehabilitation is a low-cost, vital component of both short and long term management of COPD [3,29]. Currently, the most common method of delivering pulmonary rehabilitation is a centre-based approach, which may be accomplished within an outpatient hospital department, community centre or private practice [52]. The lack of access to this intervention for a significant proportion of people with COPD is likely to be a contributing factor to the patterns of decline in exercise capacity and increase in symptoms characteristic of this disease [3]. In comparing the two modes of delivery on important clinical features and the economic impact to both the patient and the healthcare system, further information on the feasibility of a home-based model of pulmonary rehabilitation will be obtained.

It has been well documented that the effects of pulmonary rehabilitation, including those conducted via a centre-based approach, diminish over time, with deterioration in both exercise capacity and HRQOL within 6 to 12 months of program completion [53]. One key reason for this decline may be the difficulty in maintaining regular exercise routines and adherence over the longer term [53]. A critical goal of pulmonary rehabilitation is to both maximize and sustain benefits. It is possible that undertaking pulmonary rehabilitation within the home environment may promote more effective integration of exercise routines into daily life over the longer term. With greater adherence to exercise, the initial benefits of pulmonary rehabilitation may be better maintained.

The results of this study will inform guidelines for the provision of pulmonary rehabilitation in COPD and may have a significant impact on the delivery of healthcare to people with COPD. If it is demonstrated that home-based exercise achieves equivalent clinical outcomes and is cost effective compared to a hospital-based program, this increases the options for the provision of this intervention and may assist in overcoming the most frequently identified barriers to pulmonary rehabilitation.

### Trial status

Patient recruitment commenced in October 2011 and is continuing.

## Abbreviations

COPD: Chronic obstructive pulmonary disease; QALYs: Quality-adjusted life years; FEV1: Forced expiratory volume in one second; FVC: Forced vital capacity; 6MWT: 6-minute walk test; 6MWD: 6-minute walk distance; HRQOL: Health-related quality of life; SF-36: Short form-36; PRAISE: Pulmonary rehabilitation adapted index of self-efficacy; HADS: Hospital Anxiety and Depression Scale; MBS: Medicare Benefits Scheme; PBS: Pharmaceutical Benefits Scheme; SF-6D: Short form-6.

## Competing interests

The authors declare that they have no competing interests.

## Authors’ contributions

AEH, CFM, AM designed the trial protocol. AEH, AM, CFM procured the study funding. AEH and ALL drafted the manuscript and AM, CJH, ALL, ATB, RM, CH, PO, NSC, AL and CFM contributed to the manuscript. All authors read and approved the final manuscript.

## Pre-publication history

The pre-publication history for this paper can be accessed here:

http://www.biomedcentral.com/1471-2466/13/57/prepub
